# USING MIXED EFFECTS GROWTH MODEL TO EXAMINE LONG-TERM COURSE OF PHYSICAL ACTIVITIES AMONG POSTSTROKE PATIENTS

**DOI:** 10.1093/geroni/igae098.2407

**Published:** 2024-12-31

**Authors:** Ling Han, Geske Luzum, Heather Allore, Torunn Askim, Xiangchun Tan, Pernille Thingstad, Asta Håberg, Ingvild Saltvedt

**Affiliations:** Yale University School of Medicine, New Haven, Connecticut, United States; Norwegian University of Science and Technology, Trondheim, Nord-Trondelag, Norway; Yale University, New Haven, Connecticut, United States; Norwegian University of Science and Technology, Trondheim, Sor-Trondelag, Norway; Norwegian University of Science and Technology, Trondheim, Sor-Trondelag, Norway; Norwegian University of Science and Technology, Trondheim, Sor-Trondelag, Norway; Norwegian University of Science and Technology, Trondheim, Sor-Trondelag, Norway; Norwegian University of Science and Technology, Trondheim, Sor-Trondelag, Norway

## Abstract

Restoring function and regaining physical activity (PA) is a fundamental goal of post-stroke rehabilitation programs, yet empirical evidence is lacking for long-term PA. Using data from the Nor-COAST study, we followed 277 individuals (mean age at baseline: 70.1, SD 10.9). Using a thigh-worn accelerometer, PA was measured for total upright time (UPT) in minutes over each 24-hr period for 6 consecutive days at 3-, 18-, and 36-months post-stroke. A mixed effects linear growth model examined post-stroke PA course, with a patient-specific random intercept to account for nesting effects of repeated UPT measurements. On average the UPT times declined by 0.61 min (95% Confidence Interval (CI): 0.84-0.62) per month after adjusting for ten a priori selected confounding factors. The adjusted average UPT is 42.5 min (95% CI: 67.2-18.1) shorter in men than in women; while one-yr aging reduced UPT time by 22.9 min (95% CI: 35.3-10.5). As expected, the day-1 NIH Stroke Scale (mean 2.8, SD 4.3) was associated with shorter UPT time (Mean difference: 7.3 min (95% CI: 3.2-11.3)). The final adjusted model revealed substantial between-patient UPT variations (intercorrelation coefficient, 0.55). These findings suggest that the PA of post-stroke patients appear to follow a modest, yet declining course over the 3 years, with substantial variation across individuals with different demographic and clinical profiles, calling for extending professional follow-up and individually tailored PA rehabilitation programs. Future studies may need to consider broader contexts of enabling PA of patients and their families, such as “walkable” neighborhoods and socioeconomic environments of communities.

